# Corneal Deposit of Ciprofloxacin after Laser Assisted Subepithelial Keratomileusis Procedure: A Case Report

**DOI:** 10.1155/2010/296034

**Published:** 2010-06-16

**Authors:** Giacomo De Benedetti, Andrea Brancaccio

**Affiliations:** ^1^Hospital Quirón Donostia, 7, Parque Alcolea, 20012 San Sebastian, Spain; ^2^Istituto di Chimica del Riconoscimento Molecolare (CNR) c/o Istituto di Biochimica e Biochimica Clinica, Università Cattolica del Sacro Cuore, Largo Francesco Vito 1, 00168 Roma, Italy

## Abstract

*Purpose*. To report one case of corneal antibiotic deposition after ciprofloxacin administration in Laser Assisted Subepithelial Keratomileusis (LASEK). *Methods*. One case of post-LASEK treatment resulted in corneal precipitates and poor wound healing. Debris was analyzed with dark field microscopy and placed on a blood-agar plate seeded with a susceptible stain of *Staphylococcus aureus* (ATCC 29213). *Results*. The alterations resolved with discontinuation of ciprofloxacin treatment, although some residual deposits persisted subepithelially for 6 months. Analysis of precipitates revealed polydisperse crystalline needles of 183 *μ*m average length (SD = 54 *μ*m) and the excised precipitate demonstrated a zone of inhibition. *Conclusions*. Fluoroquinolone antibiotic drops have been used extensively in postsurgical treatment of refractive surgery. Corneal precipitates have been previously reported in the literature, but up to now nothing has been documented after LASEK. Polypharmacy during refractive surgery may impair epithelialisation, and clinical management should reduce toxic environment and promote ocular surface stability when performing surface ablations.

## 1. Introduction

Topical fluoroquinolones may be used as antibacterial treatment and have been associated with corneal deposits [1–4] but nothing has been reported in surface laser ablations. One case of corneal deposits occurring after the use of topical ciprofloxacin in post-LASEK treatment is presented.

## 2. Materials and Methods

A 59-year-old man opted to be submitted to a LASEK procedure in December 2008. His best-corrected visual acuity (BCVA) in the right eye (RE) was 20/25 with +3,75 sph = +0,50 cyl at 30° and in the left eye (LE) was 20/25 with +3.25 sph. His maculas showed some alteration in the retinal pigmented epithelium (RPE). The LASEK procedure was carried out bilaterally according to Dr. Camellin guidelines, applying Mytomicin C (MMC) 0.02% for two minutes at the end of the the laser-ablation [1].

At the end, lidofilcon contact lenses (Actifresh 400 – Hydron – Hamble, UK) were placed to facilitate epithelial adhesion.

Ciprofloxacin 0.3% four times/day (Oftacilox drops – Alcon Cusi – El Masnou, Spain) was started two days earlier, while the postsurgical treatment resulted in

- Ciprofloxacin 0.3% (as above),
- Diclofenac 0.1% four times/day (Diclofenaco Lepori monodosis – Angelini – Barcelona, Spain),
- Polividone 5% ad libitum (Oculotect monodosis – Novartis – Barcelona, Spain).

Debris from the deposit was analyzed with dark field microscopy and placed on a blood-agar plate seeded with a susceptible stain of *Staphylococcus aureus *(ATCC 29213). A standardized laboratory disk of ciprofloxacin containing 5 g/mL served as a control [3, 4].

## 3. Results and Discussion

The follow-up during the three days after LASEK was regular, so the patient was appointed to remove contact lenses (CL) after three days.

At the scheduled visit, his CLs were not “in situ”, although he could not explain when or how he had lost them.

**Figure 1:**
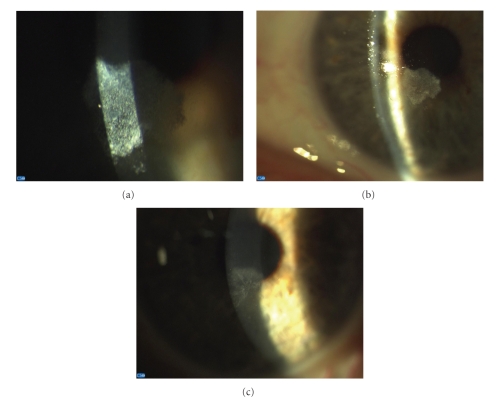
Shows the microcistic “caramel-like” opacity of subepithelial, paraoptic, inferior corneal stroma with epithelial discontinuities as appeared in the beginning (a, b) and after discontinuation of ciprofloxacin (c).

His LE showed a microcistic “caramel-like” opacity of subepithelial, paraoptic, inferior corneal stroma with epithelial discontinuities, without any sign of ocular or periocular inflammation (Figures 1(a) and 1(b)).

His RE showed only minor epithelial defects.

Administration of ciprofloxacin and diclofenac in his LE was stopped and substituted with tobramycin 0.3% (Tobrex – Alcon Cusi – El Masnou, Spain) and dexamethasone 0.1% (Dexafree monodosis – Thea – Barcelona, Spain) six times/day.

On the following day, RE too showed initial opacities of the inferior cornea and was moved to the same treatment of LE, which was gently scraped to analyze the debris as indicated in Section 2.

After four days the gross opacity in LE disappeared, although some minor subepithelial deposits and punctiform desepithelisation still persisted.

RE showed no more peculiar findings.

Tobramycin was then stopped bilaterally and Dexamethasone was tapered in 2 weeks, switching to Fluorometolone 0.1% (FML drops – Allergan – Westport, Ireland) three times/day, tapered in 6 weeks bilaterally.

During the whole period the IOP did not show any hypertensive spike.

After one month, LE opacity was very similar to a vertex keratopathy (Figure 1(c)), leading to a subepithelial inferior nubecula persisting for 6 months, with no influence on his best-uncorrected vision acuity (BUCVA), which is 20/30, nor on his BCVA (20/25 with +0.50 sph).

Deposits were confirmed to be ciprofloxacin by dark field microscopy.

The analysis of the images obtained with this technique revealed the precipitates to be polydisperse crystalline needles of 183 *μ*m average length (SD = 54 *μ*m).

The excised precipitate resulted in a zone of inhibition that measured 22.0 mm on ATCC 29213 plate; the control disk measured 17.0 mm after 24 hours.

## 4. Conclusions

Ciprofloxacin, a second-generation fluoroquinolone, is extensively used in bacterial keratitis, owing to its ease of availability, broad spectrum of activity, and lack of toxicity. Although there are many reports of crystalline corneal deposits occurring with the use of topical ciprofloxacin 0.3% and norfloxacin 0.3% [1–7], nothing has been reported after refractive surgery.

In “in vitro” tear model, drug concentration of ciprofloxacin determined a rapid precipitation that starts at 8′ postdose, producing turbidity and a significant decline in soluble drug concentration [4].

Scuderi et al. in 2003 [8] incubated rabbit corneal epithelial cells with ofloxacin at different concentrations (1.5, 3, and 6 mg/mL) and found in all the conditions tested a statistically significant dose- and time-dependent reduction in cell viability even after 8 hours. A large case-series of ofloxacin deposits after microbial keratitis has been presented by Mitra et al. in 2007 [9].

Moreover gatifloxacin, a fourth-generation fluoroquinolone, has been reported to cause intrastromal crystalline deposits with a compromised corneal epithelium, in a similar manner to ciprofloxacin [10].

Although the use of ciprofloxacin alone seems to cause physicochemical changes in the tear film, combination therapy and other factors probably contribute to alter the pH thus causing or accelerating the precipitation process.

Such picture could be supported by our case, where LE was more affected than RE but further studies are needed to understand if CL loss, presurgical antibiotic treatment, LASEK technique itself, or the application of MMC could have affected the re-epithelialisation in this case.

Accumulation of large numbers of dead cells and an altered general morphology may be further factors influencing the formation of deposits.

With reference to the case here described, at the time of presentation to the corneal service, this patient had been treated with persistent, preserved and unpreserved topical drops. 

Some of these topical medications (Oftacilox and Tobrex) contain benzalkonium chloride as preservative, which is known to disrupt cell walls by emulsifying membrane lipids, while decreasing epithelial microvilli, corneal wetting and inhibiting cell motility and surface healing [11].

It is probable that the pH balance had been altered owing to the interaction of multiple medications which, in the presence of a compromised ocular surface and of a nonhealing epithelial defect (due to the loss of the contact lenses), could result in ciprofloxacin precipitation and relative appearance of corneal deposits.

Discontinuation of ciprofloxacin and modification of topical therapy should aid the resolution of these deposits in most cases, as well as the promotion of epithelialisation, and the reduction of corneal and conjunctival toxicity. 

If deposits persist and prevent epithelialisation, they should be debrided to permit the cornea to epithelialise, as in this case, without any adverse outcome, although some deposits persisted subepithelially despite prolonged topical cortisone therapy.

In any case, for the future it is hoped that more topical drugs are going to be available as unpreserved and/or with higher pH stability, in order to reduce at the lowest level any possible complication after local treatment, especially in refractive surgery where patients are very demanding for fast outcomes.
